# Design and Feasibility of an Intervention to Support Cancer Genetic Counselees in Informing their At-Risk Relatives

**DOI:** 10.1007/s10897-016-9948-7

**Published:** 2016-04-22

**Authors:** Eveline de Geus, Willem Eijzenga, Fred H. Menko, Rolf H. Sijmons, Hanneke C. J. M. de Haes, Cora M. Aalfs, Ellen M. A. Smets

**Affiliations:** 1 Department of Medical Psychology, Academic Medical Centre, University of Amsterdam, Meibergdreef 9, 1105 AZ Amsterdam, The Netherlands; 2 Cancer Family Clinic, Netherlands Cancer Institute, Amsterdam, The Netherlands; 3 Department of Clinical Genetics, University Medical Centre Groningen, University Groningen, Groningen, The Netherlands; 4 Department of Clinical Genetics, Academic Medical Centre, University of Amsterdam, Amsterdam, The Netherlands

**Keywords:** Cancer genetic counseling, Motivational interviewing, Family communication

## Abstract

Cancer genetic counselees receive individualized information regarding heightened risks and medical recommendations which is also relevant for their at-risk relatives. Unfortunately, counselees often insufficiently inform these relatives. We designed an intervention aimed at improving counselees’ knowledge regarding which at-risk relatives to inform and what information to disclose, their motivation to disclose, and their self-efficacy. The intervention, offered by telephone by trained psychosocial workers, is based on the principles of Motivational Interviewing. Phase 1 of the intervention covers agenda setting, exploration, and evaluation, and phase 2 includes information provision, enhancing motivation and self-efficacy, and brainstorming for solutions to disseminate information within the family. Fidelity and acceptability of the intervention were assessed using recordings of intervention sessions and by counselee self-report. A total of 144 counselees participated. Psychosocial workers (*n* = 5) delivered the intervention largely as intended. Counselees highly appreciated the content of the intervention and the psychosocial workers who delivered the intervention. In the sessions, psychosocial workers provided additional and/or corrective information, and brainstorming for solutions was performed in 70 %. These results indicate that this intervention is feasible and warrants testing in clinical practice. For this, a randomized controlled trial is currently in progress to test the intervention’s efficacy.

## Introduction

Information on possible heightened risks for developing hereditary cancer, after completion of cancer genetic counseling, has implications for both the counselees and their at-risk relatives. Based on the results of a possible DNA test and counselees’ family cancer history, counselees receive individualized medical recommendations. These recommendations may include risk reduction surgery, regular screening, and DNA testing of relatives (Stichting Opsporing Erfelijke Tumoren and Vereniging Klinische Genetica Nederland Werkgroep Klinische Oncogenetica 2005; Menko et al. 2013). However, at-risk relatives for whom this information might also be of medical benefit do not always receive this information (Peterson et al. 2003; Claes et al. 2003; MacDonald et al. 2007). Thus, they lack the opportunity to make a well-informed decision regarding whether to attend genetic counseling, DNA testing and/or surveillance activities. The possible barriers playing a role in the family communication process are manifold and complex, and have been reviewed by Chivers Seymour et al. (2010) and Wiseman et al. (2010).

Current Dutch guidelines ( 2012) state that counselees and clinical geneticists are responsible for optimal dissemination of information to the family, in the case of hereditary and familial cancer syndromes for which effective preventive interventions are available, in line with international recommendations (Godard et al. 2006). It is recommended that, during cancer genetic counseling, counselees be informed about the possible implications of genetic testing for their relatives. As part of the genetic counseling, counselees receive a letter which summarizes and reaffirms the information provided during the counseling sessions. In hereditary cancer syndromes, in which a pathogenic mutation has been detected, a separate family letter is provided to be distributed by the index patient to all at-risk family members. This letter provides information on the diagnosis within the family, as well as the possibilities regarding DNA testing and preventive options.

Although counselees report feeling responsible about disclosing information to at-risk relatives (Hughes et al. 2002; McGivern et al. 2004; Wiseman et al. 2010), they may face several barriers in communicating hereditary cancer risk information to their relatives; these barriers can be categorized as: 1) lack of knowledge, i.e., a limited understanding of which family members ought to be informed about what, 2) motivation due to the desire to protect the relative or oneself e.g., from negative emotions, and 3) self-efficacy, i.e., feeling unable to inform relatives because one may not be able to reach them or does not feel confident about informing them correctly (Claes et al. 2003; MacDonald et al. 2007; Wilson et al. 2004; van den Nieuwenhoff et al. 2007). The counselees report a need for information and support in communicating genetic risk information to others (Hayat et al. 2012; Ratnayake et al. 2011). Counselees also express that their lack of confidence in disseminating information may be improved by more professional backup (Suthers et al. 2006).

Recent interventions aimed at improving disclosure of hereditary risk include the provision of an educational tool (Kardashian et al. 2012), enhanced cancer genetic information and an extra consultation with a specialist nurse who specifically addresses family communication (Roshanai et al. 2009), communication skills training (Montgomery et al. 2013), or additive counseling focusing specifically on discussing at-risk information with relatives (Forrest et al. 2008). In the two controlled studies, the interventions showed positive results on satisfaction, but not on family communication (Montgomery et al. 2013; Roshanai et al. 2009). A cohort study found an increase in the proportion of relatives attending genetic counseling services. Although it was assumed that this increase was due to improved family communication, this was not specifically investigated (Forrest et al. 2008).

We designed a counseling intervention targeted at assisting counselees in informing their at risk relatives as an addition to the genetic counseling services already provided in the Netherlands by genetic counselors. The intervention is based on the most important principles of Motivational Interviewing (MI) (Rollnick et al. 1999) and addresses the above-mentioned barriers to accurate dissemination of hereditary risk within families, with the aim to increase the efficacy of the intervention. We describe the design of the intervention, its fidelity (i.e., the degree of confidence that it can be delivered as intended) and its acceptability to counselees.

## Methods

### Design of the Intervention

The additional counseling intervention, offered by telephone and performed by trained psychosocial workers, was timed to follow-up on counselees’ receipt of their summary letter from the department of Clinical Genetics. The intervention is provided by telephone to also reach counselees who are unlikely to visit the clinic for additional counseling because of limited knowledge or motivation to inform relatives.

#### Content of the Intervention

This intervention was based on MI, a directive, client-centered counseling style for eliciting behavior change by helping clients to explore and resolve ambivalence (Miller and Rollnick 2002a; Rollnick et al. 1999). In this specific case, ambivalence between on the one hand feeling responsible to inform relatives and on the other hand the wish to protect oneself and/or their relatives form negative emotions or feeling unable to correctly inform relatives. The principles of MI take into account the healthcare professionals’ challenge of stimulating counselees to provide correct genetic cancer information to at-risk relatives, while at the same time respecting counselees’ possible wish not to inform, i.e. their autonomy (Hodgson and Gaff 2013). The format of our additional counseling session was based on a MI counseling intervention originally designed for general practitioners to improve medication adherence (Broers et al. 2005).

Two phases were distinguished within our intervention: 1) an exploratory phase, including agenda setting, exploring, and evaluation, and 2) a motivational counseling phase including information provision, building motivation and self-efficacy, and brainstorming.

##### Phase 1

This phase starts with agenda setting, meaning that the psychosocial worker introduces the purpose of the conversation (i.e. to discuss family communication related to hereditary cancer risk) and checks whether the counselee is comfortable with doing this. The aim of agenda setting is to introduce the subject without evoking resistance. If the counselee states that he or she is not willing to talk about the subject of family communication, the psychosocial worker will accept this refusal and ‘roll with resistance’, one of the general principles of MI (i.e. by confirming counselees’ autonomy and express understanding) (Miller and Rollnick 2002b).

Then, the counselees’ current and planned pattern of informing relatives is systematically explored. Psychosocial workers are instructed to systematically verify for each relative whether the counselee is aware whether the relative has to be informed and what information to disclose. They have the pedigree and summary letter available, and can use these as ‘gold standard’ of the information that needs to be disclosed. In this way, psychosocial workers gain insight into counselees’ level of knowledge. By means of exploration, the psychosocial worker aims to understand counselees’ perspectives, motives and (possible) resistance to inform relatives. Psychosocial workers do this in a reflective, supportive manner to reduce resistance and increase motivation to inform at-risk relatives. Perceived barriers to disclose information to relatives are discussed. Next, the psychosocial worker evaluates whether or not the counselee has informed all at-risk relatives according to the information stated in the summary letter. If the counselee has informed all at-risk relatives properly, the psychosocial worker ends the counseling session; if not, the psychosocial worker proceeds to the second phase.

##### Phase 2

The first element of this phase is to provide additional and/or corrective information, as required. For example, if a pathogenic BRCA1/2 mutation is found it is important to stress that male relatives also need to be informed, as they can also inherit the risk and pass it on to their children. Or, in case a counselee does not have sufficient knowledge regarding which relatives are at-risk, the psychosocial worker may point out that an already informed relative is not eligible for DNA testing or surveillance measures while another, not informed, relative is at-risk and should be informed. The ‘elicit-provide-elicit model’ (Rollnick et al. 2008) is used: first eliciting the person’s understanding and information needs, then providing this information neutrally, followed by inviting the counselee to interpret the information.

Another element of the second phase is to build counselees’ motivation and perceived ability to correctly and sufficiently inform relatives, i.e., their self-efficacy. In case a counselee did not inform all at-risk relatives, the psychosocial worker invites the counselee to verbalise arguments in favor of informing relatives to reinforce these arguments and thus strengthen the counselees’ motivation. Similarly, the counselees’ self-efficacy is assessed and strengthened. Psychosocial workers are trained to react to and accept any type of reaction of the counselee in order to promote honest answers and reduce resistance to inform relatives.

As a final element, the psychosocial worker invites counselees to engage in active brainstorming on possible solutions for their experienced barriers in informing at-risk relatives, with the aim to help counselees develop a plan. To motivate and enhance counselees’ self-efficacy, counselees are encouraged to list possible solution themselves rather than only hearing the solutions from the psychosocial workers. Finally, on request of both the counselees and psychosocial workers, a second supportive session can be provided.

#### Psychosocial Workers

Five psychosocial workers, with a BA degree in social work and additional training and experience in genetics, were trained to deliver the intervention. In the Netherlands, psychosocial workers are consulted in standard genetic care in case of difficulties in decision-making, family or social environment, living with cancer, general emotional problems, or problems affecting children. Therefore, these psychosocial workers are experienced in providing psychosocial support and have more time for this kind of specialized counseling than clinical geneticists and/or genetic counselors. For the purpose of the intervention, psychosocial workers had access to counselees’ medical files and had the counselees’ pedigree available; consequently, because they are well informed, this saves the counselee the bother of discussing medical issues yet again.

#### Training of the Psychosocial Workers

The psychosocial workers attended two days of training provided by two senior communication trainers (psychologists), with experience in MI (e.g., Broers et al. 2005), who collaborated in the development of the intervention. Before the first meeting, the psychosocial workers received a manual which guides the intervention and explains the two-phase model. This manual was written by the primary researcher in collaboration with the two trainers. During the first training day, the trainers provided background information on MI during an interactive presentation. They explained the mechanisms of patient resistance towards advice and how to successfully deal with such resistance. Counselees’ autonomy and the issue of responsibility about informing relatives received special attention. The interactive character of the theory presentation and the ensuing group discussions aimed to create insight into the principles of MI, clarify possible problems, share insights, and induce an attitude change in the psychosocial workers, if required.

Target skills, for example agenda setting, exploration of ambivalence, and dealing with resistance, and so called ‘practitioner traps’, i.e. understandable yet unprofessional reactions in response to counselees reluctance to inform relatives, were addressed by means of role playing. During the first meeting a professional actor played a prepared ‘paper’ case and, during the second meeting, the actor played a counselee who the psychosocial workers found ‘difficult to handle’. The psychosocial workers discussed their objectives related to the different elements of the intervention and all psychosocial workers gave and received feedback from the group and the trainers.

Additionally, counseling sessions of each psychosocial worker were audio-recorded and the content was coded (see paragraph on measures). The results were used for feedback during the second training session to improve psychosocial workers’ performance.

After each training day the psychosocial workers received a generic summary of the discussed ‘practitioner traps’ (e.g., confronting, advising, stressing the importance of informing relatives) and ‘recommendations’ (e.g., respect counselees’ autonomy, do not ‘go faster’ than the counselee, be aware that the counseling has not failed if the counselee makes a well-informed decision to not inform certain relatives).

### Assessment of Fidelity and Acceptability of the Intervention

#### Participants

Consecutive counselees who had an appointment at the department of Clinical Genetics of three Dutch university hospitals were eligible to participate if they: 1) were the first in their family to visit the Clinical Genetics department (i.e., index patients), 2) received counseling for hereditary or familial colon or breast and/or ovarian cancer, 3) had at least one relative at increased risk that was eligible for genetic testing and/or surveillance, 4) were aged 18 years and over, and 5) were able to read and write Dutch. Counselees could either have received a conclusive DNA test result (i.e., a pathogenic mutation has been found), or an inconclusive test result (i.e. no mutation or a variant of unknown significance was found), or DNA testing was not indicated or not performed for other reasons (e.g., patient’s wishes, relative deceased). Nevertheless, participating counselees with an inconclusive result, or without a DNA test, had an elevated cancer risk based on their family history. It is common policy to offer counselees a DNA test if the chance of an onco-genetic mutation is at least 10 %.

#### Procedure

The present study was part of a larger parent investigation testing the psychometric properties of the Informing Relatives Inventory (IRI), a battery of instruments used to measure counselees’ knowledge, motivation, and self-efficacy regarding the disclosure of hereditary cancer risk information to at-risk relatives; results of that study are presented elsewhere (Geus de et al. 2015). Participants did not receive any form of reimbursement.

Upon receipt of their summary letter, counselees were invited by their clinical geneticist or counselor to participate via an information letter, an informed consent form, a return slip, and an invitation for a web-based or paper baseline questionnaire (T1), if preferred. Participants who provided informed consent were contacted within one week to schedule the telephone counseling session with the psychosocial worker. This session was audio-taped. After the intervention the counselees received a second questionnaire (T2) by mail.

The present study was exempted from formal approval by the Medical Ethics committee of the Academic Medical Center in Amsterdam, since the Medical Research Involving Human Subjects Act (WMO) does not apply.

#### Measures

The sociodemographic characteristics of the counselees were assessed in the baseline questionnaire. Clinical characteristics were extracted from the departments’ summary letter and counselees’ medical records.

**Fig. 1:**
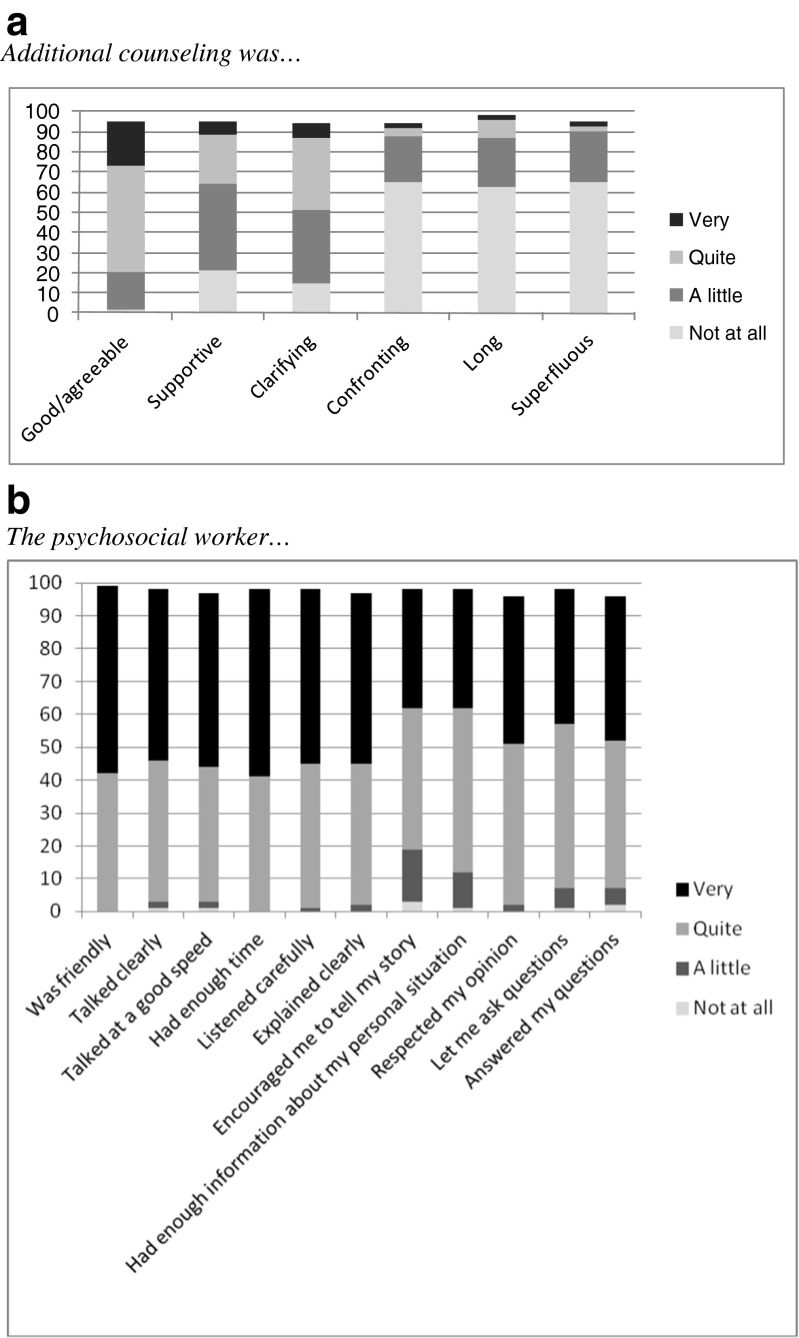
**a** Percentage of counselee’s (n-121) endorsing 6 items to evaluate the additional counseling using a 4-point rating scale. **b** Percentage of counselee’s (n-121) endorsing 11 items to evaluate the psychosocial worker using a 4-point rating scale

At T2, counselees completed a 27-item questionnaire developed for the purpose of this study. This included six items on the *fidelity* of the intervention, using a 4-point Likert scale (1 = not at all, 4 = very much). For example: “Did the psychosocial worker discuss the pros and cons of informing relatives?”, or “Did the psychosocial worker motivate you to inform at-risk relatives?”. Counselees’ *acceptability* of the intervention was assessed with 6 items addressing the quality of the intervention (1 = not at all, 4 = very much) (Fig. 1a); 11 items evaluating psychosocial workers’ communications (1 = not at all, 4 = very much) (Fig. 1b), 3 items concerning practical issues, such as the intervention being delivered by telephone (7-item response scale 1 = very problematic, 7 = not at all problematic), the time between the last counseling session and the intervention (too short, just right, too long), and the need for an additional session (yes, no), and an overall rating (“How would you rate your consultation with the psychosocial worker”; 1 = very bad, 10 = excellent).

To further assess whether the psychosocial workers delivered the intervention as intended, counseling sessions were content coded from the audio-tape using a self-developed coding scheme based on an existing instrument (Wiggers et al. 2005). Table 2 shows the items which were coded as ‘present’ or ‘not present’. A random selection of six consultations was coded separately by one researcher (EdG), and also by a research assistant who was not part of the research team. As agreement was almost 100 %, all remaining consultations were content coded by the research assistant alone. The duration of each consultation was also assessed.

## Results

### Participants

Of the 358 eligible counselees invited to participate, 228 responded of which 74 (32 %) declined participation, resulting in a response rate of 43 % (*n* = 154). A total of 144 participants completed the baseline questionnaire (T1), 139 participants received the intervention, and 121 counselees completed the second questionnaire (T2). Participants did not differ from non-participants regarding gender, age, disease type and carrier status (Table 1).

**Table 1:** Demographic characteristics of the study sample (*n* = 144)

	Participants	Non-participants (n = 210)	p-value
Age in years: mean (SD)	54 (12)	52 (13)	0.29
Range	18–80	23–83	
Sex			0.11
Male	44 (31 %)	48 (23 %)	
Female	100 (69 %)	162 (77 %)	
Education level^a^			
Low	21 (15 %)		
Middle	65 (45 %)		
High	50 (35 %)		
Marital status			
Married/Partner	121 (84 %)		
Widowhood	6 (4 %)		
Single	16 (11 %)		
Counseled for			0.42
Breast and/or ovarian cancer	63 (44 %)	101 (48 %)	
Colon cancer	81 (56 %)	109 (52 %)	
Carrier status			0.13
Mutation	20 (14 %)	18 (9 %)	
Inconclusive result^b^	124 (86 %)	192 (91 %)	
Previously diagnosed with			
Breast and/or ovarian cancer	47 (33 %)		
Colon cancer	37 (26 %)		
Other cancer	19 (13 %)		
No cancer diagnosis	49 (34 %)		

^a^Low: non/primary school, middle: secondary/lower level vocational school, high: college/university (8 missing)

^b^Including counselees who were not tested (8 participants and 10 non-participants)

### Fidelity of the Intervention

Five psychosocial workers provided the intervention (ranging from 8 to 41 sessions per psychosocial worker). Three counselees had a second session with the psychosocial worker, because both agreed that one session was insufficient to cover all issues at stake.

### Self-Reported (6 items)

Of the counselees, 54 % reported that the intervention had clarified which relatives they should inform. Counselees who received the second phase of the intervention (*n* = 29) reported that in 73 % of the cases the pros and cons of informing relatives were discussed, 72 % of counselees reported that the psychosocial worker addressed difficulties in informing relatives, and 65 % reported that they had jointly brainstormed how to inform relatives. A total of 59 % of the counselees felt motivated by the psychosocial worker to discuss genetic risk information with relatives, and 73 % felt that the psychosocial worker had enhanced their confidence to inform relatives correctly.

### Audio-Recording

Audio recordings of 131 sessions were analyzed. In five cases the additional counseling was not recorded and the quality of three was insufficient. The mean duration of the sessions was 27 (range 8–82, SD 13.7) min. Results of the coding of the sessions are presented in Table 2.

**Table 2:** Checklist (27 items ^a^^,^^b^) to assess on the basis of audiorecorded consultations (*n* = 131) whether psychosocial workers (*n* = 5) delivered the two-phased intervention as intended

**Phase**	**Element**		**After 1st training day** (*n* = 79)	**After 2nd training day** (*n* = 52)
1	1	**Agenda setting**		
		Is the aim of the counseling explained?	92 %	96 %
		Does the counselee give permission for the counseling?	75 %	98 %
		Does the psychosocial worker ask whether or not the counselee has received the summary letter?	80 %	81 %
		Does the psychosocial worker explain she has pedigree and summary letter?	82 %	96 %
	2	**Exploring**		
		Did the psychosocial worker ask whether the counselee was advised to inform relatives?	4 %	8 %
		Did the counselees identify him/herself which relatives are at-risk/need to be informed?	41 %	73 %
		Did the counselee report him/herself which advise the relatives should get?	44 %	69 %
		Is the risk to develop cancer for relatives discussed?	54 %	85 %
		Did the psychosocial worker systematically ask for each at-risk relative?	90 %	94 %
		Did the psychosocial worker make clear to the counselee whether all relatives were correctly informed or not?	80 %	94 %
	3	***Evaluation*** **(** ***relatives not informed*** **)**	*n* = *19* (*24* %)	*n* = *16* (*31* %)
2*	1	**Information provision**		
		Did the psychosocial worker ask whether the counselee is willing to continue talking about informing relatives?	6 %	18 %
		Did the psychosocial worker add information?	61 %	82 %
		Did the psychosocial worker correct information?	11 %	29 %
		Did the psychosocial worker check whether or not the counselee understood the information?	11 %	12 %
	2	**Building motivation and self-efficacy**		
		Did the psychosocial worker ask the counselee to list arguments not to inform relatives?	44 %	53 %
		Did the psychosocial worker ask whether or not the counselee was willing to list arguments in favor of informing relatives?	28 %	35 %
	3	**Brainstorming**		
		Did the psychosocial worker stimulate the counselee to actively brainstorm about informing relatives?	72 %	77 %
		Was there brainstorming?	72 %	65 %

*Percentages are calculated from the number of counselees that have not informed all their relatives

^a^Study specific checklist based on Wiggers et al. 2005

^b^All audiotapes coded by a research assistant

#### Phase 1

Agenda setting was performed correctly by the psychosocial workers; they explained the aim of the additional counseling, and asked for permission to talk about the subject of informing relatives properly. Only in 5 % of the sessions did the psychosocial workers explore whether the counselee knew whether he/she has been advised to inform at-risk relatives by the department of Clinical Genetics. After the first training session, in only half of the sessions did the counselees themselves identify which relatives were at risk and which information they should receive; this information was often presented by the psychosocial worker. After the second training session, the psychosocial workers more often explored counselees’ degree of this knowledge. It was concluded from the audiotapes, that in 27 % percent of the sessions the psychosocial workers assumed that the counselees had not yet correctly informed their at-risk relatives, and therefore proceeded to phase 2 of the intervention.

#### Phase 2

Information giving was highly prevalent in the second phase; the psychosocial workers added information in 82 % of the sessions. However, they seldom checked whether or not the counselee understood the information (11 %). Psychosocial workers contributed to building motivation to disclose risk information by asking the counselee to list arguments not to inform relatives in almost half of the sessions, and to list arguments in favor of informing relatives in a third of the sessions. Brainstorming about ways to inform their relatives was performed in about 70 % of the sessions (Table 2).

#### Acceptability of the Intervention

Data on counselee’s evaluation of the intervention and the psychosocial worker are provided in Fig. 1a and 1b, respectively.

The majority of counselees reported that they were not bothered that the additional counseling was by telephone, only three counselees did mind this. The mean time between the summary letter and the intervention was 40 (range 14–159, SD 24.3) days. The timing was considered good by 85 % and too long by 14 %. A minority of 5 % of the counselees would have liked another appointment with the psychosocial worker. The counselees graded the additional counseling with an average score of 8 (SD 1.2).

## Discussion

We developed a brief intervention as an adjunct to usual care, to assist counselees in making an informed decision about disclosing hereditary risk information to relatives and to act on this. The intervention focuses on i) enhancing counselees’ knowledge of which relatives to disclose important genetic-related information to, ii) exactly what information should be disclosed, and ii) increasing their motivation and self-efficacy in informing their at-risk relatives. The intervention appeared to be widely accepted by participating counselees; both the content and practicality were highly rated.

The intervention is grounded in MI, a well-known clinical method with an encouraging evidence base for its efficacy (Miller and Rollnick 2009). The choice for MI as the guiding model for the intervention is based on the MI tenet that it is important to elicit the person’s own inherent arguments for relevant health behavior, rather than imposing someone else’s (Miller and Rollnick 2009). In line with this view, the psychosocial workers adopted a collaborative role and promoted a well-informed decision about which relative to inform about what, rather than to determine whom the counselee ‘should’ inform. The psychosocial worker has to facilitate the counselee’s ability to make a conscious decision about the communication of hereditary risk, with an awareness of what affects this decision and how relatives are subsequently informed. Thus, the goal of the intervention is to address the process of informing relatives. Although this process may result in more relatives being informed, this was not the primary goal of our intervention. Our approach to the dissemination of hereditary risk information within families via the counselee might be considered as ‘indirect’ or ‘passive’, as opposed to direct contact between the genetic counselor and relatives. Although the latter may result in more relatives being informed and seeking genetic counseling, a direct approach raises issues about counselees’ confidentiality, the relative’s right to privacy and the right not to know.

Counselees evaluated the additive counseling as not being confronting, long, or superfluous. On the other hand, over half of the counselees did not experience the counseling as clarifying and/or supportive. These might be the counselees who had already informed their relatives and who experienced no problems in this respect. However, although the intervention did not provide them with additional support, they seemed to appreciate the offer to discuss family communication, as indicated by the high rates for usefulness, pleasantness and for the way in which the psychosocial workers delivered the intervention. In sum, although not all counselees may have benefitted, the intervention did not appear to cause them any harm.

It is noteworthy that less than half of the eligible counselees accepted the invitation to participate in this study. Other Dutch psychosocial studies in the field of Clinical Genetics reported comparable response rates (Albada et al. 2012; Vos et al. 2011) suggesting that these generally healthy users of this medical service are less likely to contribute to research than populations of (chronically) ill patients. Nevertheless, the limited uptake suggests that, for the majority of counselees, additional counseling in discussing hereditary risk with relatives does not serve a high need for support.

Analysis of the audio-recorded consultations leads us to conclude that many elements of the intervention were delivered as intended. Thus, the MI approach seems feasible for experienced psychosocial workers, after appropriate training. Agenda setting and exploring which relatives were informed was carried out very systematically. Not all elements of the second phase were prevalent in the audio-taped consultations; however, this is in line with the manual which states that one or more of these elements should be used only if a counselee did not correctly inform their relatives. It is nevertheless noteworthy that, of the counselees who had not yet informed all relatives at-risk, 41 % reported that the psychosocial worker had done little to motivate them to inform their relatives. This supports the finding that psychosocial workers infrequently used the motivation-enhancing strategy to have the clients themselves list what is good about informing relatives, suggesting room for improvement in this respect. Also, the psychosocial workers rarely checked whether counselees’ had understood the information they had provided.

The training for psychosocial workers comprised two sessions, with ample opportunity for practice in between sessions and for obtaining individualized feedback based on audio-recorded consultations; this appears to have been beneficial. Initially, psychosocial workers were inclined to present information themselves concerning which relatives needed to be informed and what information should be provided, rather than to explore counselees’ knowledge on whom to inform about what. Increased exploration of counselees’ knowledge as a result of the second training session appears to have led to an increase in the detection of counselees’ who had not yet informed all at-risk relatives and, consequently, in more frequent additional or corrective information. This finding underscores the importance of a thorough exploration to obtain proper understanding of the counselees’ knowledge level.

Only in a minority of consultations was a second phase needed to correct/add information and build counselee’s motivation and self-efficacy to further inform relatives. Although there are many examples of problematic risk communication within families, our results tentatively suggest that many counselees succeed in disseminating risk information.

### Practice Implications

Results of this feasibility study show that psychosocial workers who are required to assist in the process of communication of genetic information within families can acquire and apply the skills necessary for a theory-based approach such as MI. We also demonstrated that counselees who consent to a conversation about family communication, appreciate the psychosocial workers’ approach. Therefore, this intervention appears to be feasible for clinical practice. We emphasize, however, that in families with hereditary or familial cancer, we recommend that the subject of informing relatives be considered an integral part of the genetic counseling process and be addressed in the first counseling session (Menko et al. 2013). We do not yet know whether the intervention is effective, i.e., improves counselees’ ability to make a well-informed decision about whether or not to inform relatives about their possible heightened risk and preventive options, and to what extent they feel better equipped to do so. If found to be effective, a telephone intervention is more likely to be implemented than a face-to-face type of intervention.

### Research Recommendations

Currently, we are conducting a randomized controlled trial, based on a new study sample, to test the efficacy of this additional telephone intervention in clinical practice (Geus de et al. 2014). We hypothesize that the intervention will improve counselees’ knowledge, motivation and self-efficacy to inform their at-risk relatives. In addition, we will investigate whether the intervention leads to more relatives being informed by the counselee. If efficacious, implementation of this relatively brief intervention may be warranted.
